# Clinical outcomes of drug-coated balloon for treatment of de novo coronary artery disease with and without diabetes

**DOI:** 10.15537/smj.2022.43.12.20220534

**Published:** 2022-12

**Authors:** Zhaoqian Zhang, Qiang Tan, Jiarui Zhang, Xinhui Wang, Qian Wang

**Affiliations:** *From the Department of Cardiology, Qinhuangdao First Hospital, Hebei Medical University, Hebei, China.*

**Keywords:** drug-coated balloon, percutaneous coronary intervention, coronary artery disease, diabetes mellitus

## Abstract

**Objectives:**

To retrospectively evaluate the efficacy of drug-coated balloon (DCB) in patients with de novo coronary artery disease with and without diabetes.

**Methods:**

Patients with de novo coronary artery and undergoing percutaneous coronary intervention (PCI) with DCB were enrolled from March 2018 and March 2020, including 312 patients being divided into the diabetes group (n=110), pre-diabetes group (n=48) and non-diabetes group (n=154). The primary endpoint was major adverse cardiac events (MACE) (MACE; cardiovascular death, non-fatal myocardial infarction, target lesion revascularization, and target vessel revascularization) at 24 months.

**Results:**

In diabetes group, the incidence of MACE at 24 months (19.1%) was higher than in the non-diabetes group (12.5%) and pre-diabetes group (10.4%) (*p*<0.05). Cox regression revealed that diabetes (HR [hazard ratios] 2.049, 95% CI 1.056-4.284), bifurcation lesion (HR 5.255, 95% CI 2.765-9.986), Syntax score (HR 1.098, 95% CI 1.040-1.559) and hemoglobin A1c (HR 1.084, 95% CI 1.160-1.741) were independent predictors of MACE in patients performing PCI with DCB (all *p*<0.05). However, pre-diabetes did not increase the risk of MACE (HR 1.560, 95% CI 0.542-4.490, *p*>0.05).

**Conclusion:**

Diabetes increased the risk of MACE in patients performing PCI with DCB.

**D**iabetes mellitus (DM) is a prevalent disease all over the world. It is estimated that the number of patients with DM will be at least 592 million cases in 2035.^1^ Coronary artery disease is one of the most important complications of DM. Although the use of drug-eluting stents (DES) decreased the rates of restenosis in patients with DM, diabetic patients still have an increased risk of adverse events after percutaneous coronary intervention (PCI) than patients without diabetes.^2^ Previous studies demonstrated that the risk of stent thrombosis was 25%-80% higher in diabetic patients than in non-diabetic patients.^3,4^ In-stent restenosis (ISR) was more common in patients with DM than patients without DM.^5^ Pre-diabetes is an early condition of diabetes with impaired glucose tolerance or impaired fasting glucose.^6^ Because pre-diabetes is a reversible condition, the influence of pre-diabetes on clinical outcomes of patients performing PCI is unclarity.

It has been testified that drug-coated balloons (DCB) are effective in treatment for de novo coronary artery disease.^7,8^ Compared to drug-eluting stents, DCB has some special advantages, such as the absence of metal stent and rapid delivery of Paclitaxel.^9^ Theoretically, due to these benefits, DCB therapy in diabetic patients would have comparable results to non-diabetic patients. However, few studies comparing clinical outcomes in patients with DM, pre-DM and normoglycemia who performed PCI with DCB.

The aim of this study was to compare the effect of DCB on major adverse cardiac events (MACE) in patients of de novo coronary artery disease with DM, pre-DM or without DM.

## Methods

A retrospective cohort study was carried out on enrolled patients who received PCI with DCB between March 2018 and March 2020. The inclusion criteria: i) patients aged at least 18 years with stable angina pectoris, unstable angina pectoris, and non-ST segment elevation myocardial infarction (NSTEMI); ii) Patients received DCB treatment in de novo coronary artery. While the exclusion criteria: i) Patients with ISR; ii) Unprotected left main lesion; iii) Prior coronary artery bypass grafting (CABG); iv) Heavily calcified in culprit vessel; v) Residual stenosis no less than 30% after balloon pre-dilation or C-type dissection after balloon dilation.

Diagnosis of diabetes and pre-diabetes were based on American Diabetes Association definition and diagnosis of diabetes mellitus.^10^ Diabetes, fasting plasma glucose test (FPG) ≥7.0 mmol/L or glycated hemoglobin A1C (HbA1C) ≥6.5%; pre-diabetes, 5.5 mmo/L ≤FPG <7.0 mmol/L or 5.7% ≤HbA1C) <6.5%.

This study was approved by the ethics committee and informed consent had been obtained from the study participants prior to study commencement.

Similar to the methods employed in our previous study (Tan et al^11^), interventional procedures were performed. Patients underwent coronary angiography and PCI using transradial or transfemoral approach. The decision to perform PCI with DCB or DES depended on the recommendation of the interventional cardiologist. A quantitative coronary angiography (QCA) system (GE QCA, Centricity AI 1000) was conducted to analyze reference diameter, lesion length, pre-procedure minimal lumen diameter (MLD) and post-procedure MLD.

Pre-dilated balloon was used to perform pre-dilation of the target vessel before DCB treatment. The inflation time for DCB was 30-40 seconds with an overlap of ≥2 mm on each edge of the pre-dilatation balloon-treated segment.

The DCB (Bingo; Yinyi company, China) was covered with a surface area of 3 µg paclitaxel/mm^2^ and ranged from 15 mm to 30 mm in length and 2.0 mm to 3.5 mm in diameter.

All patients were administered 100 mg aspirin daily and received 75 mg Clopidogrel daily for at least 3 months. The average duration of diabetes was 27±22 months, the types of medication were insulin (54.6%), metformin (61.3%), SGLT-2 inhibitor (37.1%), GLP-1 receptor agonist (27.3%), DPP-4 inhibitor (25.3%), Alpha Glucosidase Inhibitor (31.3%). Patients underwent clinical observation at clinic for 24 months. Clinical follow-up was carried out at 1 month, 6 months, 12 months, and 24 months. Blood examination and electrocardiogram were performed during follow-up. Angiography follow-up was performed 9-12 months after DCB procedure (angiography follow-up at 9-12 months after the procedure was routinely advised by physicians, which was not triggered by angina or other symptoms).

The primary endpoint of this study was incidence of combined MACE of 24 months, defined as cardiovascular death, non-fatal myocardial infarction (MI), target lesion revascularization (TLR), and target vessel revascularization (TVR). Elevation of serum troponin I to 3 times the upper limit of normal with chest pain lasting more than 30 minutes was defined as MI.^11^ Target lesion revascularization was defined as any repeat revascularization due to restenosis of the DCB-treated lesion (both proximal and distal to the treated segment beyond 5 mm). Target vessel revascularization was defined as any repeat revascularization of the DCB treated vessel.^11^

### Statistics analysis

The SPSS Statistics for Windows, (Version 17.0. Chicago: SPSS Inc.) was used to do statistical analyses. Continuous variables were expressed as mean ± standard deviation of the mean, and compared using one-way Anova; χ^2^ statistics or Fisher exact test was used in Categorical variables. Kaplan-Meier method was conducted to estimate incidence of MACE of 24 months. Cox proportional hazards regression analysis was used to estimate the hazard ratios (HR) and its 95% confidence intervals (CI) of MACE. A probability value <0.05 was considered statistically significant.

## Results

The enrolled patients were divided into 3 groups, DM group (n=110), pre-DM group (n=48) and non-DM group (n=154), with baseline clinical characteristics shown in Table 1. There were no difference in age, gender, family history, hypertension, smoking, previous myocardial infarction, and clinical presentation among the 3 groups. Glucose levels, triglycerides and HbA1C were higher in diabetic patients than in pre-diabetes group and non-diabetes group. Pre-diabetic patients also had higher levels of glucose and HbA1C than non-diabetic patients. Other laboratory characteristics such as left ventricular ejection fraction (LVEF), cholesterol, low density lipoprotein-cholesterol, high density lipoprotein-cholesterol, and homocysteine had no significantly difference among the 3 groups.

**Table 1:** - Clinical characteristics of patients with diabetes, pre-diabetes and non-diabetes (N=312).

Characteristics	diabetes (n=110)	Pre-diabetes (n=48)	Non-diabetes (n=154)	*P*-value
Age	62.41±9.84	60.51±9.58	60.80±9.79	0.105
Gender (M/F)	66/44	33/15	113/41	0.071
Current smoker	47 (42.7)	18(37.5)	64(41.6)	0.826
Family history	23 (20.9)	13(27.1)	30 (19.5)	0.529
Hypertension	51 (46.4)	21(43.8)	74 (48.1)	0.867
Prior stroke	7 (6.4)	4 (8.3)	9 (6.0)	0.828
Prior myocardial infarction	6 (5.5)	3 (6.3)	7 (4.5)	0.880
LVEF (%)	66.09±6.67	61.21±9.45	65.69±6.98	0.122
LVD (mm)	47.60±3.92	49.46±4.05	48.64±4.73	0.209
LA (mm)	38.26±5.24	39.08±7.81	36.951±5.85	0.005
***Clinical presentation***				0.629
Stable CHD	24	7	29	
Unstable angina	65	30	101	
NSTEMI	21	11	24	
TC (mmol/l)	4.38±1.13	4.52±1.13	4.37±1.02	0.786
TG(mmol/l)	2.54±1.61	1.95±0.91	1.68±0.99	0.009
LDL-C(mmol/l)	2.45±0.86	2.52±0.85	2.44±0.77	0.894
HDL-C(mmol/l)	1.04±0.21	1.07±0.23	1.08±0.23	0.550
Creatinine(µmol/l)	62.53±15.42	64.76±18.86	65.72±15.43	0.370
Glucose (mmol/l)	6.98±2.06	6.66±0.54	5.15±0.45	0.000
HbA1c (%)	6.75±0.92	6.14±0.19	5.24±0.35	0.000
Urine acid (mmol/l)	334.36±89.80	332.10±97.24	332.16±94.21	0.987
HCY (mmol/l)	15.79±9.92	15.32±7.17	18.06±14.72	0.559
BMI	26.01±3.38	25.90±3.05	24.96±2.92	0.065

Values are presented as number and percentages (%). LVEF: left ventricular ejection fraction, LVD: left ventricular diameter, LA: left arterial diameter, NSTEMI: non-ST segment elevation myocardial infarction, TC: cholesterol, TG: Triglyceride, LDL-C: low density lipoprotein-cholesterol, HDL-C: high-density lipoprotein cholesterol, HCY: homocysteine, BMI: body mass index, HbA1c: hemoglobinA1c, CHD: coronary heart disease

As shown in Table 2, the target artery, Syntax score, the rate of bifurcation and multi-vessel disease had no significant difference among the 3 groups. However, diabetes group had more numbers of diseased vessels than pre-diabetes group and non-diabetes group. Quantitative coronary angiography analysis showed that reference vessel diameter, pre-procedure MLD, and post-procedure MLD were bigger in non-diabetes group than in diabetes group and pre-diabetes group. Diabetic patients had longer lesion length and DCB length than non-diabetic patients and pre-diabetic patients.

**Table 2:** - Angiographic and procedural characteristics of diabetes, pre-diabetes and non-diabetes patients with DCB (N=312).

Characteristics	Diabetes (n=110)	Pre-diabetes (n=48)	Non-diabetes (n=154)	*P*-value
***Target artery***				
Left anterior descending	47/110 (42.7)	19/48 (39.6)	62/154 (40.3)	0.900
Diagonal	17/110 (15.5)	8/48 (16.7)	13/154 (8.4)	0.134
Left circumflex	19/110 (17.3)	9/48 (18.8)	34/154 (22.1)	0.614
Right coronary artery	27 /11(27.5)	12/48 (25.0)	45/154 (29.2)	0.664
Bifurcation	31/110 (28.2)	13/48 (27.1)	51/154 (33.1)	0.729
Multivessel disease	61/110 (55.5)	25/48 (52.1)	79/154 (51.3)	0.795
Number of diseased vessels	2.48±0.77	2.14±0.79	2.08±0.84	0.001
Syntax score	12.81±5.30	12.95±4.71	12.50±4.72	0.851
***Target lesion***				
Reference diameter (mm)	2.57±0.21	2.57±0.24	2.64±0.19	0.047
Lesion length (mm)	20.46±9.60	17.37±6.83	18.49±8.15	0.042
Diameter stenosis (%)	91.07±7.01	90.87±7.33	89.68±8.23	0.271
Pre-procedure MLD (mm)	0.58±0.41	0.59±0.34	0.61±0.42	0.036
Post-procedure MLD (mm)	2.54±0.19	2.55±0.36	2.61±0.13	0.024
***The characteristics of DCB***				
Diameter (mm)	2.61±0.14	2.62±0.11	2.63±0.13	0.067
Length (mm)	22.53±11.76	20.75±10.15	20.62±12.41	0.012

MLD: minimal luman diameter, DCB: drug-coated balloon

**Figure 1:**
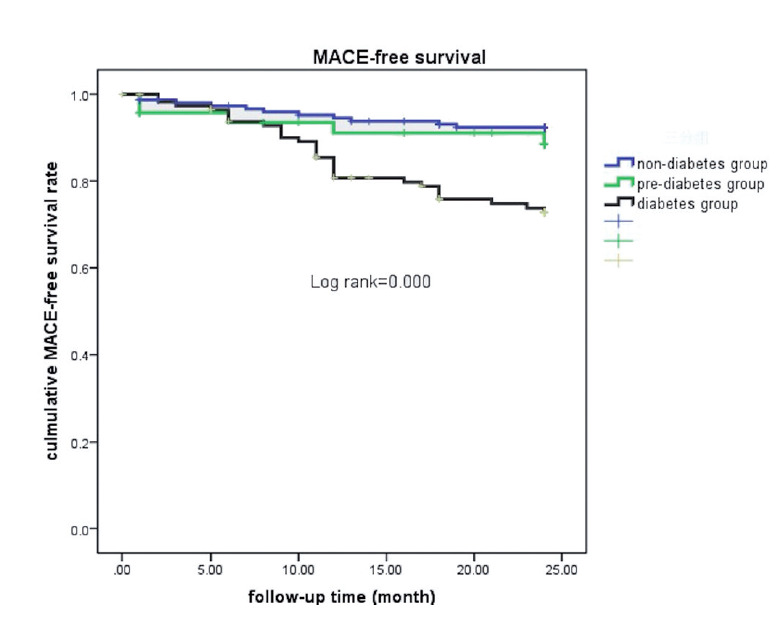
- Major adverse cardiac events (MACE)-tree survival in patient performing drug coasted balloon

Survival analyses by Kaplan-Meier method showed a poor prognosis in diabetes group with a higher incidence of MACE at 24 months compared to non-diabetes group and pre-diabetes group (Figure 1). But there were no difference between pre-diabetes group and non-diabetes group. As shown in Table 3, the incidence of the primary endpoint in diabetic patients was significantly higher than that in non-diabetic patients and pre-diabetic patients (*p*<0.05), which was mainly driven by the increase in TVR and TLR. However, the incidence of cardiovascular death, non-fatal MI had no significant difference among the 3 groups during follow-up.

**Table 3:** - Clinical outcomes of diabetes and non-diabetes patients with drug-coated balloon (N=312).

Outcomes	Diabetes (n=110)	Pre-diabetes (n=48)	Non-diabetes (n=154)	*P*-value
All cause MACE	21 (19.1)	6 (12.5)	16 (10.4)	0.026
Cardiovascular death	1 (0.9)	0(0)	1 (0.6)	0.331
Non-fatal MI	5 (4.5)	2(4.2)	6 (3.9)	0.805
TLR	12 (10.9)	4(8.3)	7(4.5)	0.047
TVR (including TLR)	15(13.6)	4(8.3)	9 (5.8)	0.025

Values are presented as number andpercenatges (%). MACE: major adverse cardiac events, MI: myocardial infarction, TLR: target lesion revascularization, TVR: target vessel revascularization

The baseline clinical and angiographic characteristic of patients, stratified by MACE, are summarized in Table 4. Patients with MACE had higher levels of glucose and HbA1C than patients without MACE. Syntax score, numbers of diseased vessels and the rate of bifurcation were higher in MACE group. Patients with MACE also had longer lesion length and DCB length than patients without MACE.

**Table 4:** - Clinical characteristics of patients with MACE or without MACE (N=312).

Characteristics	MACE group (n=43)	Non-MACE (n=269)	*P*-value
Age	61.54 ± 10.87	61.21 ±9.66	0.841
Gender (M/F)	31/12	182/87	0.562
Current smoker	21(48.8)	113 (42.0)	0.401
Family history	14 (32.6)	52 (19.3)	0.821
Hypertension	24 (55.8)	132 (49.1)	0.412
Prior myocardial infarction	5 (11.6)	11 (4.1)	0.030
Prior stroke	6 (14.0)	14 (5.2)	0.037
Diabetes	21 (48.8)	79 (29.4)	0.011
Pre-diabetes	9 (20.9)	39 (14.5)	0.278
***Clinical presentation***			0.927
Stable angina	7 (16.3)	49 (18.2)	
Unstable angina	27 (62.8)	169 (62.8)	
NSTEMI	9 (20.9)	51 (19.0)	
TC (mmol/L)	4.33±1.31	4.43±1.61	0.681
TG (mmol/L)	2.51±1.61	1.93±1.59	0.104
LDL-C (mmol/L)	2.50±1.06	4.41±0.02	0.768
HDL-C (mmol/L)	2.37±0.73	1.93±1.19	0.942
Glucose (mmol/L)	6.51±1.59	2.45±0.75	0.025
Hemoglobin A1c (%)	6.35±0.99	1.06±0.23	0.000
Creatinine (µmol/L)	64.92±21.43	5.94±1.53	0.872
Urine acid	341.82±133.56	5.83±0.88	0.585
HCY (mmol/L)	16.56±7.28	55.18±15.27	0.833
Body mass index	26.07± 3.49	331.39±84.38	0.198
LVD (mm)	48.68 ± 4.67	48.42±4.41	0.759
LAD (mm)	39.30±8.01	37.70±5.79	0.185
LVEF (%)	64.65±9.78	65.25±6.70	0.625
***Target artery***			
Left anterior descending	11(25.6)	117 (43.5)	0.027
Diagnal	7(16.3)	31 (11.5)	0.295
Left circumflex artery	6(14.0)	56 (20.8)	0.376
Right coronary artery	19(44.2)	65 (24.2)	0.006
Bifurcation	25 (58.1)	70 (26.0)	0.000
Multivessel disease	31 (72.1)	134 (49.8)	0.007
Number of diseased vessel	2.67±0.71	2.16±0.82	0.000
Syntax score	16.18±4.45	12.07±4.82	0.000
***Target lesion***			
Reference diameter (mm)	2.68±0.15	2.71±0.21	0.475
Lesion length (mm)	21.46±8.80	18.64±8.46	0.049
Diameter stenosis (%)	92.93±6.14	90.01±7.78	0.019
Pre-procedure MLD (mm)	0.58±0.15	0.59±0.16	0.738
Post-procedure MLD (mm)	2.49±0.19	2.48±0.24	0.692
***The characteristics of DES***			
Diameter (mm)	2.62±0.21	2.61±0.21	0.729
Length (mm)	26.51±12.89	21.81±11.57	0.016

LVEF: left ventricular ejection fraction, LVD: left ventricular diameter, LAD: left arterial diameter, NSTEMI: non-ST segment elevation myocardial infarction, TC: cholesterol, TG: Triglyceride, LDL-C: low density lipoprotein-cholesterol, HDL-C: high-density lipoprotein cholesterol, HCY: homocysteine, MLD: minimal luman diameter, DCB: drug-coated balloon, MACE: major adverse cardiac events, DES: drug-eluting stents, M/F: male/female

As Table 5 showed, the results of Cox regression revealed that diabetes (HR 2.049, 95% CI 1.056-4.284), bifurcation lesion (HR 5.255, 95% CI 2.765-9.986), Syntax score (HR 1.098, 95% CI 1.040-1.559) and HbA1C (HR 1.084, 95% CI 1.160-1.741) were independent predictors of MACE in patients performing PCI with DCB (all *p*<0.05).

**Table 5:** - Proportional hazards model regression of major adverse events in patients undergoing PCI with DCB.

Characteristics	Univariate analysis Coefficient (95% CI)	*P*-value	Multiple analysis Coefficient (95% CI)	*P*-value
Diabetes	2.348 (1.142-4.829)	0.020	2.049 (1.056-4.284)	0.047
Pre-diabetes	1.560 (0.542-4.490)	0.560	---	---
Bifurcation	5.265 (3.077-9.066)	0.000	5.255 (2.765-9.986)	0.000
Syntax score	1.138 (1.089-1.189)	0.000	1.098 (1.040-1.559)	0.001
DCB length	1.033 (1.015-1.050)	0.011	1.028 (1.006-1.051)	0.072
DCB diameter	1.475 (0.277-7.860)	0.646	---	---
Post MLD	1.138 (0.350-3.703)	0.829	---	---
Reference diameter	0.606 (0.143-2.577)	0.059	1.730 (0.4545-4.393)	0.422
Lesion length	1.041(1.014-1.069)	0.002	1.870(1.202-2.909)	0.076
BMI	1.064 (0.960-1.179)	0.238	---	---
Glucose	1.153 (1.002-1.327)	0.046	1.103 (1.001-1.377)	0.151
Hemoglobin A1c	1.522 (1.190-1.948)	0.001	1.084 (1.160-1.741)	0.014
Number of vessel diseased	2.793 (1.937-4.033)	0.000	1.321 (0.864-2.081)	0.198
Age	0.993 (0.959-1.129)	0.711	---	---
Hypertension	1.135 (0.571-2.038)	0.139	---	---
Smoke	1.131 (0.570-2.245)	0.724	---	----
Prior MI	0.950 (0.670-1.348)	0.775	---	---
LDL-C	1.018 (0.656-1.580)	0.936	----	---

DCB: durg-coated balloon, Post MLD: post-procesure minimal luman diameter, LDL-C: low density lipoprotein-cholesterol, MI: myocardial infarction

## Discussion

The main finding of this study was that clinical outcomes were poorer in diabetic patients treated with DCB, compared to non-diabetic patients. This finding indicated that diabetes increased the incidence of MACE in patients performing PCI with DCB.

The number of patients with diabetes and coronary artery disease is increasing rapidly worldwide. It is reported that patients with DM compose 25% to 30% of all patients undergoing coronary artery revascularization.^12^ Several studies had testified that diabetic patients treated with new-generation DES remained at higher risk of adverse events following PCI. BIO-RESORT study reported that diabetes increased one year risks of mortality and repeat revascularization after treatment with DES.^13^ Korea Acute Myocardial Infarction Registry (KAMIR) study found a higher 2-year incidence of stent thromboses in patients with diabetes compared to patients without diabetes.^14^ However, the association between pre-diabetes and adverse outcomes after PCI has not been clearly established. A combined analysis of BIO-RESORT and BIO-NYX showed that pre-diabetes increased 3 years MACE rate in patients performing DES.^15^ Kim et al^14^ compared MACE rate of patients with diabetes or pre-diabetes after successful performing PCI with second generation DES. The results showed that incidence of MI in the pre-diabetes group was significantly lower than that of the diabetes group.^16^

One of the beneficial feature of DCB is the local delivery of paclitaxel to coronary artery without leaving metal sten.^17^ As a result, DCB decreased the incidence of vessel thrombosis after PCI compared with DES implantation. Lots of studies have shown that DCB is effective in the treatment of in stent restenosis (ISR) and de novo lesion, especially in small vessel disease.^17,18^ However adverse events risk in patients with diabetes treated with DCB had not been fully assessed. Until now, only one research has discussed outcomes of DCB in diabetic patients. Pan et al^17^ reported that diabetic patients had higher TLF and TLR rates following DCB angioplasty without a substantial increase in the risk of MACE, cardiac death, myocardial infarction, and revascularization.

In this observational study, we evaluated the outcomes of PCI with DCB in diabetic patients versus non-diabetic patients and pre-diabetic patients, suggesting diabetic patients treated with PCI with DCB exhibited a higher incidence of MACE, TLR and TVR than non-diabetic patients. However, the incidence rates of cardiac death and MI were comparable in the 3 groups. These findings enhance our understanding of the high risk of diabetes in patients with PCI. We did not find different MACE rate between pre-diabetes patients and non-diabetes patients, pre-diabetes was not an independent predictor of MACE in this current study.

Previous studies found that patients with DM have more diffuse and complex CAD than non-diabetic patients, which is also consistent with the current study that patients with diabetes had a smaller diameter of the coronary artery, longer lesions and more serious lesions.^17-19^ These researches demonstrated that CAD in the presence of DM has unique characteristics. The high risk of MACE in patients with diabetes may be secondary to the complex pathophysiological mechanisms, including endothelial dysfunction, chronic inflammation, and activation of platelet.^18^ Our previous study found that diabetes impaired the functions of endothelial progenitor cells (EPC) which play a key role in maintaining endothelial function.^20,21^ EPC dysfunction leads to defects of endothelium repairment and vascular complications in diabetic patients.^22^ Inflammation is another mechanism of diabetes-induced vascular remodeling and progression of adverse myocardial diseases.^23^ Platelet activation and atherosclerotic thrombosis are increased in diabetic patients compared to non-diabetic patients.^10^

### Study limitations

The current study has several limitations. First, it was a single-center study, the sample size was relatively small. Second, it is a retrospective and observational but not a randomized controlled study. More research is required to determine how DCB affects diabetic people.

In conclusion, our findings suggested that diabetic patients experience higher MACE, TVR and TLR rates upon DCB angioplasty with compared to non-diabetic patients. The risk of cardiac mortality and MI, however, was not significantly increased by DM. To demonstrate the effectiveness of DCB in diabetic patients, additional research and effort are still required.
